# Impact of Memory Problems Post-stroke on Patients and Their Family Carers: A Qualitative Study

**DOI:** 10.3389/fmed.2020.00267

**Published:** 2020-06-19

**Authors:** Eugene Y. H. Tang, Christopher Price, Blossom C. M. Stephan, Louise Robinson, Catherine Exley

**Affiliations:** ^1^Population Health Sciences Institute, Newcastle University, Campus for Ageing and Vitality, Newcastle upon Tyne, United Kingdom; ^2^Division of Psychiatry and Applied Psychology, Institute of Mental Health, School of Medicine, University of Nottingham, Innovation Park, Nottingham, United Kingdom

**Keywords:** stroke, cognition, qualitative, primary care, dementia

## Abstract

**Background:** Memory problems post-stroke are common and for some, these problems could then progress to a dementia illness. Once in the community, stroke-survivors are looked after by their family doctors although there is evidence that these patients may struggle to access appropriate help in the community for these problems. Although a stroke-survivor may be physically capable of performing daily tasks, they and their families may have to learn to manage and adapt to their new memory deficits. There is often less focus on cognitive recovery post-stroke from clinical services perhaps because of the lack of awareness and evidence of these adaptations. There is also good evidence that organized stroke care improves physical recovery but no equivalent evidence for the effectiveness of cognitive rehabilitation. The aim of this qualitative study was to report the impact of memory problems on the stroke-survivor and their family once they are living in the community.

**Methods:** Semi-structured interviews were conducted with patients and family carers to gain an in-depth understanding of their experiences. Participants were invited to take part in an interview at around six and 12-months post-stroke. A topic guide was developed to explore participant's care experiences post-stroke when they have also presented with memory difficulties. Data collection and analysis were iterative; all transcripts were anonymized. The data were thematically analyzed.

**Results:** Twenty-two interviews were conducted. Five family carers and ten stroke-survivors were interviewed at six-months post-stroke, of these eight stroke-survivors and four family carers agreed to a 12-month follow-up interview. They identified several areas of impact: (1) impact on daily life; (2) emotional impact; and (3) compensating strategies implemented in response to impact.

**Conclusion:** Living with stroke combined with memory impairment can have negative effects on the stroke-survivor and their family once in the community. Health professionals and services in the community need to recognize the burden of managing symptoms post-stroke for these individuals and their families. Understanding the impact can enable more effective community and specialist support to be provided particularly if we were to also identify those who may then be at risk of a future dementia illness.

## Introduction

Following a stroke and discharge from specialist services into the community, patients are expected to navigate through complex health systems and treatment regimens whilst recovering (1). However recovery in the community involves not only dealing with physical aspects, but the psychological and emotional impact of the stroke, to enable the individual to rebuild and restructure their world (2). For some stroke-survivors the “unseen” emotional changes post-stroke can be more disabling than the physical impairments (3).

Post-stroke cognitive deficits are common (4) and add to the challenges already faced by stroke-survivors. In terms of current services, primary and secondary healthcare professionals have already identified a lack of clarity when managing stroke individuals with memory difficulties (5). Similarly, patients provide accounts of fragmented care (1), which could have a negative impact on the stroke-survivor and their families. Although there have been calls for stroke clinicians to have increased awareness of the cognitive consequences of stroke (6), even if this happens, services presently available to support patients and families are limited. A previous survey by the Stroke Association found that 77% of stroke survivors have problems with memory yet nearly 50% of stroke-survivors reported that the support they received for their memory problems and fatigue was poor (7). The recent NHS Long Term Plan has specifically highlighted the need for stroke rehabilitation, which is better integrated into community care for the longer term (8). In order for this to be achieved, there needs to be greater awareness of post-stroke cognitive changes particularly in primary care where these patients will be discharged to.

Stroke is known to be a strong independent risk factor for dementia (9). In fact, around 10% of individuals will develop dementia soon after their first stroke and around a third will be affected after recurrent stroke (10). Compared to the general population, the incidence of dementia is nearly 50 times higher in the year after a major stroke (11). However, it is clear that for those with memory problems post-stroke, they have obstacles in accessing primary healthcare services (12). A recent systematic review has highlighted the dissatisfaction amongst stroke-survivors and caregivers with the lack of proactive follow-up, which includes primary care (13). It is not only marginalization that leads to this sense of abandonment but these patients also do not have the skills to re-engage with services (13). It has become increasingly apparent that the role of primary care is to provide this continuity of care for these patients, particularly if we also wish to identify those at the greatest risk of developing a future dementia illness. Highlighting the “hidden” impact of reduced cognitive performance following stroke could assist clinicians in understanding the variation in difficulties experienced and recognizing when additional assessment might be necessary.

To assess whether there is a need for additional services following discharge into the community, it is necessary to describe and highlight the daily impact of cognitive impairment post-stroke. The aim of these qualitative interviews was to seek understanding of the scale and nature of impact of memory problems for stroke-survivors and their families in the community.

## Methods

### Design and Setting

Qualitative semi-structured interviews were conducted with community dwelling stroke-survivors and their family carers from the North-East of England. Older stroke-survivors (aged over 60 years old) who presented to their six month post-stroke specialist review and expressed any subjective memory concern following their stroke were invited to participate in the study. If applicable, their family carers were also invited to participate. If people were interested in taking part, their contact details were passed onto the research team. Purposive sampling was used to ensure that a range of experiences could be captured i.e. to ensure a mix of genders and a range of carers were recruited. One researcher, a medical doctor (EYHT), then contacted the individual to provide further information regarding the study and provide an opportunity for potential participants to ask questions. If they agreed, participants were asked to take part in an interview soon after their six-month stroke clinic review (baseline) or around 12 months post-stroke (follow-up) or at both time points.

### Data Collection

Semi-structured interviews were used to elicit the experiences of stroke-survivors who subsequently developed memory problems. Following a review of the literature and discussion amongst the research team an interview topic guide was created. Interviews were designed to seek participants' views on a range of topics including their experiences in stroke services, their subsequent experiences of living in the community e.g., in seeking support and information if their memory did not improve and their feeling toward risk assessment for dementia. This paper focuses on the impact of memory problems after stroke, which participants elaborated on when discussing these main topics. Other topics in this study including access to services (12) and views on risk assessment for dementia have been published separately (14). The study took an iterative approach. That is, data collection and analysis occurred concurrently, to ensure that emerging topics identified in earlier interviews could be explored in subsequent ones. Interviews were conducted between April 2016 and August 2017 in participants' place of choice (their homes) either individually or together if requested by both participants (i.e., stroke-survivor and family carer together).

### Data Analysis

All interviews were digitally audio recorded, anonymized, and transcribed verbatim. Data analysis followed the principles of the constant comparative method (15) and the data was thematically analyzed (16). One researcher (EYHT) familiarized himself with all interviews and read all transcripts line by line and coded the data to identify some initial themes. Two members of the team (EYHT and CE) went through these themes to identify areas of overlap. These themes were subsequently categorized into broader overarching themes to provide an overview of participant views. Data collection ceased when data saturation had occurred, which was defined as the point where informational redundancy had been reached (17). These themes were discussed and agreed with the wider researcher team. The data was managed on NVivo 11 software. The paper conforms to the Standards for Reporting Qualitative Research checklist (18) (Supplementary Table 1).

## Results

Twenty-two interviews were conducted in total. Ten stroke-survivors and five carers were interviewed at 6 months post-stroke, of these, a further eight stroke-survivors and four carers agreed to an interview 6 months later (see Table 1). During interviews, participants discussed in detail the impact of their memory problems following their stroke and these have been grouped into three themes.

**Table 1:** Interview participants.

**Unique identifier (patients and carers)**	**Role**	**Gender**	**Age**	**Ethnicity**	**Follow-up interview**
P1	Stroke-survivor	Female	80	Caucasian	No
P2	Stroke-survivor	Female	76	Caucasian	Yes
P3	Stroke-survivor	Female	72	Caucasian	Yes
P4	Stroke-survivor	Male	75	Caucasian	Yes
P5	Stroke-survivor	Male	80	Caucasian	Yes
P6	Stroke-survivor	Male	74	Caucasian	Yes
P7	Stroke-survivor	Female	73	Caucasian	Yes
P8	Stroke-survivor	Female	82	Caucasian	Yes
P9	Stroke-survivor	Male	84	Caucasian	No
P10	Stroke-survivor	Male	79	Caucasian	Yes
C1	Carer of P1 (Husband)	Male	79	Caucasian	No
C2	Carer of P4 (Wife)	Female	79	Caucasian	Yes
C3	Carer of P5 (Daughter)	Female	57	Caucasian	Yes
C4	Carer of P6 (Wife)	Female	71	Caucasian	Yes
C5	Carer of P8 (Daughter)	Female	60	Caucasian	Yes

### Impact on Daily Life

Stroke-survivors and their carers were asked directly how their lives had been affected by their new memory difficulties following their stroke. Stroke-survivors gave examples of general everyday activities which they had started to struggle with. This ranged from remembering important dates to doing everyday tasks both at baseline and follow-up interviews:

“*…I think it's getting worse. I had a bad day yesterday. I really felt- I call bingo at the over 60s and I've been doing it for five year and yesterday, I couldn't remember how to use the machine. I didn't switch it on right and I had to get someone to come and reset it. I just couldn't remember.” (P3 at baseline)*

“*It's funny. I've got a car, and I had a puncture, and it was funny and I knew I had a puncture, and yet when I hopped into the car the following days, and I was driving along the road, “Oh, God. I had a puncture” … I had to stop the car and see it was still flat.” (P10 at follow-up)*

In addition to discussing how everyday activities were affected, stroke-survivors often commented on the difficulties they had during conversations with others:

“*I can be in the middle of a sentence and I forget what I'm talking about, I've really got to sit and think. Emm, I can go to the shops and I'll be in the shop … I want something and when I want something but I can't think of exactly what I want. I can show them, but I cannot tell them.” (P7 at baseline)*

For this participant the conversational difficulties were still apparent at her follow-up interview:

“*Trying to make conversations at times is very hard and I get my words mixed up or I can't say them … I know they're there, but I just can't get them out, emm but the people that are around us all knows and they'll put us right, or I'll look at them and say, “What was that again?” … I'm in the middle of a conversation and then me mind just completely goes blank now. “What was I saying?” As soon as they put us right, I can pick the conversation up again as such, or I'll keep repeating myself.” (P7 at follow-up)*

It was also quite clear to family members caring for the stroke-survivor what a significant impact the memory problems had become for the individual:

“*…Taking him outside, as soon as he went out the front door, he got used to being in the house and he knew where he was in the house. Got him to the front door and he was just stopped and looking in the street. He didn't recognize the street. Emm and even when we came back he would continue walking past the house. Didn't realize he'd got home. Emm, that has improved … He's going round the block, round the estate his self sometimes.” (C3 at baseline)*

### Emotional Impact

Both stroke-survivors and their family carers reported examples of how their memory difficulties affected them emotionally. Stroke-survivors were finding their need to rely and loss of independence hard as this represented a significant change in role and challenge to their own identity:

“*It's the depending on people, which I didn't used to. I was the one that people came and said,” Dad, have you got time for to do this or do that. “Now I'm depending on them, that's, that's the part I'm finding hard.” (P5 at baseline)*

This may lead to low confidence in being able to deal with activities they would attribute to their pre-stroke selves:

“*I'm slowly getting that back; I've got bits of memory back but I haven't got sufficient to – because I've been asked to take back me chairmanship and really I'm not confident enough to do it.” (P4 at baseline)*

Carers commented on the frustration when the stroke-survivor would forget to do simple tasks:

“*Oh yeah well, I mean he doesn't seem to retain it. When I tell him something he can't retain it and I suppose if you can't take it in, you can't remember it. So that's a problem and I get frustrated sometimes and I get angry with me self for getting frustrated because I know he can't help it … [Stroke-survivor] always put like the bins out and when they were done then he would come bring them in and things like that and now I went out the other night and he hadn't brought them in and I thought, “Oh for goodness sake.”” (C4 at baseline)*

For some carers it was frustrating that the stroke-survivor seemed oblivious to the their efforts in looking after them:

“*But I have lost my temper with her a couple of times, and I have actually shouted at her and said, “You cannot do this … it's not safe for you, you cannot do it. You've got to listen and do as you're told.” She doesn't take it very easily, and the next morning she forgot it's ever happened … Then I didn't feel good, I felt terrible doing it.” (C5 at baseline)*

Families of stroke-survivors were also concerned about undermining or hurting the feelings of the stroke-survivor as they tried to deal with the difficulties encountered:

“*…He still asks the same question two or three times. Even though you've said, “We've already told you that.” And the hardest part is with wur family, because they're having to say, “We've already heard that.” Or, “You've already asked [me] that.” And they are feeling a little bit disrespectful, if you understand what I mean? (C2 at follow-up)*

However, interviews at 12-months, suggest that for some family members they had felt that things had changed for the better as there was more acceptance of the new normal:

“*But as I say, he seems to be like laughing about it more. Whereas before it was like, really, he was getting uptight about, “Oh, here we go again, I've done something stupid. I've said something stupid. I know I've said this umpteen times.” But now he takes it more sort of in his stride.” (C3 at follow-up)*

### Compensating Strategies Implemented in Response to Impact

Responding to their new memory difficulties, stroke-survivors would often adapt and find new practical ways to manage their symptoms in order to appear to function ‘normally' in the community.

“*If I'm going anywhere like that, I'd always try to arrange to put the money, if I'm like we get a taxi, now I put the money in this pocket for me taxi so I know that I've got it there. And that's what I do, I put things like ready for us just to pay out and it's not as much distraction to meself” (P1 at baseline)*

Participants would discuss the need to find ways of reminding themselves of everyday activities:

“*Like, I had an appointment for the vet for the dog and because I didn't look at the notice board, I nearly forgot it. I had to get, rush at the last minute to the vet. Eh, so, now, before I go to bed, I sit with a piece of paper and I write down the things I have to do the next day. Emm, me friend have- me and Lavender spend a lot of time together and if she's coming, or I'm supposed to go there, I always have to ring and ask her again, “What time are we going to meet?” Because I forget.” (P3 at baseline)*

Writing things down and preparation were common methods used by stroke-survivors to help them remember. This was important both to enable them to continue to function and participate in their community:

“*I don't like to do, mess anybody around like you know. What I'm doing now is I've got a calendar behind you, I write everything- every, try to write everything down now like you know so I don't forget. And em it was funny because em I had some more, I had some tests just before Christmas because our doctors, it's just local, start changing medication. And oh gosh, and with it being the end of the year and I hadn't got- Funnily enough, I didn't have a new calendar for to put the new ones on. And ey the things I was forgetting, I went- Actually I went and saw the chemist and em she em put me on the right road like you know, and different things. Even the chemist phoned me up to see how I was getting on with me medication like you know.” (P10 at baseline)*

At follow-up the same participant discussed further adaptations that he made as time had passed. These adaptations were necessary so that he would remain up to date with current events and relevant in his social circle:

“*Respondent: … but if I put the television on or I read the paper, and I read about things … now I'll go out tonight. On a Friday, I only go out on a Friday and Saturday night, you know just to have bit chinwag with me friends at the local club … I'll show you. I write it all down, most of it down and em what's going on in the football world, you know? And I write it down and I digest this before I go out tonight. ….so, I know what I'm talking about. …. Because we're talking about stuff I don't know anything about. I've missed it, you know? And I says oh I missed this, that… I don't like to be, what's the word? Pushed out, because I don't know what I'm talking about, you know.” (P10 at follow-up)*.

Carers also gave examples of how they also needed to adapt in terms of changing roles and the impact on their lives:

“*There's a complete role reversal now. I am the carer and she's now the dependant. Any medical visits, any visits to the doctors, any organization of prescriptions, anything like that I have to do.” (C5 at baseline)*

Stroke-survivors themselves also recognized the significant role their family played in their ongoing care and support:

“*I often thought, you know someone who hasn't got this, these people [family] around ya and everything, it must be very hard.” (P4 at baseline)*

“*You see, I said the other day, “I don't think I've seen my own doctor,” and she says, “Mum, she's been twice to see you.” Well, I've, I've forgotten. I can't remember that, and she says she's been twice and she says I've only got to pick up the phone and tell her and she'll be here.” (P8 at baseline)*

However, one family carer participant remarked that although she was now undertaking more tasks which many would associated with being a more formal caring role, she did not see herself as a carer and she was still very much a spouse first and foremost:

“*One of our friends suggested that I put in for a carer's allowance and then I thought, I thought I don't want to be a carer, I'm not his carer, I'm his wife. You know, I mean wives help their husbands, like husbands help wives don't they. I thought no I'm not doing that, no, no, no.” (C4 at baseline)*

## Discussion

In this study, we have been able to describe the impact of post-stroke memory problems on the stroke-survivor and their families once they are in the community. The impact can be practical or emotional, and lead to changes and adaptations in order for the stroke-survivor to continue to function in the community. Participants have mainly described compensating strategies without the input from a healthcare professional. This may mean that these deficits are invisible to their family doctors or multidiscplinary rehabilitation teams. There may also be a lack of appropriate mechanisms or pathways by which clinicians are able to recognize and support these individuals, who then took their own actions to try to reduce the impact of their cognitive difficulties. For some, these deficits persist and primary care clinicians should be aware of the longer term affects of such cognitive difficulties even when other more visible physical deficits have improved. This would enable clinicians to advise patients and their families so that they can ensure long-term support is available when necessary without the need for additional carer burden.

A recent meta-analysis found that the pooled prevalence of those with cognitive impairment one year post-stroke was 38% (19), but this may not necessarily be due to the most recent cerebrovascular event. Indeed, cognitive problems may instead reflect new identification of problems with non-stroke causes present amongst older people. For many, cognitive dysfunction may still be present even when there are minimal or no physical disabilities (20). In a study looking at domain-specific cognitive impairments 3 months following a stroke, it was found that even in cases with excellent clinical recovery (modified Rankin Scale = 0–1, no disability) 71% of participants had some cognitive impairment (21). Further, at 15-month follow-up domain-specific cognitive impairments were related to functional disability (21). There are also interactions between cognitive and mental health as perceived stress and depressive symptoms could also increase the likelihood of having subjective cognitive complaints (22). Participants in this study demonstrated the physical and emotional impact that the stroke and additional memory problems have had on their daily lives. Living with a potentially disabling chronic condition such as stroke, in addition to new cognitive deficits adds additional burden to the stroke-survivor and their families. Indeed, subjective cognitive complaints (with working memory being the most frequent) have been shown to predict difficulties with social intergration but can be mediated by depressive symptoms (23). Further, objective cognitive test performance has been found to be associated with self-reported cognitive complaints with the strongest association being found in memory (24). However, this is not always necessarily the case for example in young stroke patients (25) where cognitive metrics are less well developed or when the perceived cognitive difficulty is a reflection of psychological distress and low mood (26).

Although cognitive outcome following a stroke can vary (27), there is the risk of progression to post-stroke dementia (4). It is therefore important to be able to have systems in place to ensure these individuals receive adequate support particularly as subjective memory complaints can predict 2-year incident dementia (28). The period following their stroke and then possible transition to long-term cognitive failure or even a dementia illness itself can be extremely difficult for the stroke-survivor and their families. Looking at the dementia diagnostic journey in general, a core feature running through is in fact “living with uncertainty” (29). Outwith the context of stroke, the time from thinking something may be amiss to then looking to make contact with a healthcare professional can be as long as two and a half years for dementia (30). However, this transition to illness recognition to illness presentation is not confined to dementia alone (29) and will be applicable to stroke-survivors dealing with their post-stroke selves. Therefore stroke-survivors with memory difficulties will not only have to live with the uncertainty of their stroke but may also have to live with the uncertainty of a potential dementia illness. Even if they do not develop dementia, many of them will still need to live with persisting cognitive deficits and healthcare professionals and services need to be able to recognize the additional burden this presents to the stroke-survivor and their families.

Many stroke-survivors report unmet clinical and social needs following their stroke (31). Further, stroke-survivors and their caregivers can feel abandoned due to for example passivity of services or perhaps because they do not have the knowledge or skills to re-engage (13). Yet patients and their families still need to find ways to adapt in order to continue to function in the community. Looking to adapt to their new difficulties was found to be important in a number of ways for both sets of participants in this study. In particular, as one post-stroke participant reported, there was the need to feel that they remain relevant and not excluded in their social circles. Indeed, regaining their social and community activities following a stroke can confer a sense of confidence and connection to family and friends (32). However, previous research has also found that, when discussing quality of life, stroke-survivors often discussed changes in social relationships for example feelings of frustration from increased dependence on others (33). Similarly, “carers” interviewed for this study were family carers or informal carers i.e., they may not recognize themselves as a carer. Previous research has highlighted the importance of informal carers in the management of long term conditions (34), and also that spouse carers of individuals with other chronic conditions, such as multiple sclerosis, resist both the role and the label of a carer (35). Family carers in this study often carried out caring duties not because of any formal perceived change in role but because of their pre-existing relationship with the stroke-survivor. They did not see it as a requirement, rather a natural part of a reciprocal relationship. However, resisting the identity of a carer could mean that they are also less likely to acknowledge the need for support (36). Health professionals need to recognize that in order to enable and support a stroke-survivors social network, besides accounting for the possible impact of both physical and language disabilities, changing social needs should also be recognized (37). Primary care professionals who may have pre-existing relationship with these individuals are well placed to provide such holistic assessments and care.

The hidden impact of subjective memory deficits is not insignificant as evidenced by the fact that participants in this study had themselves often adapted according to individual circumstances. The role of primary care clinicians could be to follow-up these patients more readily post discharge from specialist stroke services with onward referral to the appropriate service. This could be tackled in two ways. First, referral for formal assessments of cognition and intervention through psychological services or memory clinic referral if applicable, second, practical assistance to support patients to continue to live well and safely in the community. However, if more formal psychological intervention for example cognitive rehabilitation is to be offered, then these services need to be made more readily available in the community. In England, neuropsychology assessment after stroke to assess cognitive difficulties and provide support is often not formally available (38). From a practical point of view, patients could also be referred to community allied health professionals for assessments and continued adaptive interventions, for example occupational therapists to enable them to adapt and manage better in their own homes. To facilitate this, there needs to be agreement and consensus across different hospital and community services about the pathway specific for this group of individuals with relevant information and practitioner training available to assist clinicians.

### Strengths and Limitations

There are several strengths to this study. We were able to capture the experiences of stroke-survivors and their families over time when adapting to their new memory problems following discharge from stroke specialist services. We do recognize some limitations. We confined the study to one area of England (i.e., the North-East) and the study participants were all Caucasian. However, services post-stroke will be similar across England and we would anticipate the experiences of others exhibiting memory deficits post-stroke would be similar. Further studies should look to assess whether there are any cultural aspects which may impact upon these individuals in addition to what has been found in this study, particularly amongst informal family carers in other ethnicities. We also recognize that we have only recruited participants who had memory deficits and other cognitive (e.g., visuospatial, attention etc) deficits have not been included. We recruited participants from stroke clinics and cognitive assessments were not performed. This meant that we could only assess those who self-reported memory disturbance as other cognitive domains were not tested for. Finally, we recognize that we only had female family carers in this study, which reflects the nature of the stroke population. As such, we are not able to elaborate whether the same issues would be found in male family carers.

## Conclusion

The addition of a cognitive problem such as memory difficulties can add significant burden to both the stroke-survivor and also their family carers. There is evidence from this study that when participant's were followed up, they do manage to compensate. This compensation often involves the family which can result in additional burden on the family carers who may not identify themselves as carers in the first place. Clinicians need to be aware that these invisible aspects of stroke can persist and that stroke-survivors and their family may require additional post-discharge support in the community. However, timely support can only be offered in the community if the appropriate services and pathways are also available to the primary care clinician. Showing evidence of need is the first step toward developing that care provision.

## Data Availability Statement

The datasets for this manuscript are not publicly available because the data are transcripts of interviews that reflect the views of individuals and complete anonymization cannot be guaranteed. Requests to access the datasets should be directed to Eugene Tang, e.y.h.tang@newcastle.ac.uk.

## Ethics Statement

The study involving human participants was reviewed and approved by London—Hampstead Research Ethics Committee (reference 16/LO/0133). The patients/participants provided their written informed consent to participate in this study. Written informed consent was obtained from the individual(s) for the publication of any data included in this article.

## Author Contributions

ET conceived the framework for this study, collected, analyzed and interpreted the data, and prepared the manuscript for submission. CP helped to conceive the framework for this study and critically evaluated the manuscript. BS helped to conceive the framework for this study and critically evaluated the manuscript. All authors contributed to the article and approved the submitted version. LR helped to conceive the framework for this study and critically evaluated the manuscript. CE helped to conceive the framework for this study and assisted with the analysis of the data and contributed to the drafting of the manuscript and also critically evaluated the manuscript.

## Conflict of Interest

LR is supported by a NIHR Senior Investigator award (NF-SI-0616-10054). The remaining authors declare that the research was conducted in the absence of any commercial or financial relationships that could be construed as a potential conflict of interest.
