# Supplementary material for: Impact of Memory Problems Post-stroke on Patients and Their Family Carers: A Qualitative Study
Source: Front Med (Lausanne). 2020 Jun 19;7:267. doi: 10.3389/fmed.2020.00267 (PMC7317277; doi:10.3389/fmed.2020.00267)
Supplement: Supplementary file 1 [file Table_1.docx]

**Supplementary Table 1: Standards for Reporting Qualitative Research Checklist^1^**

**
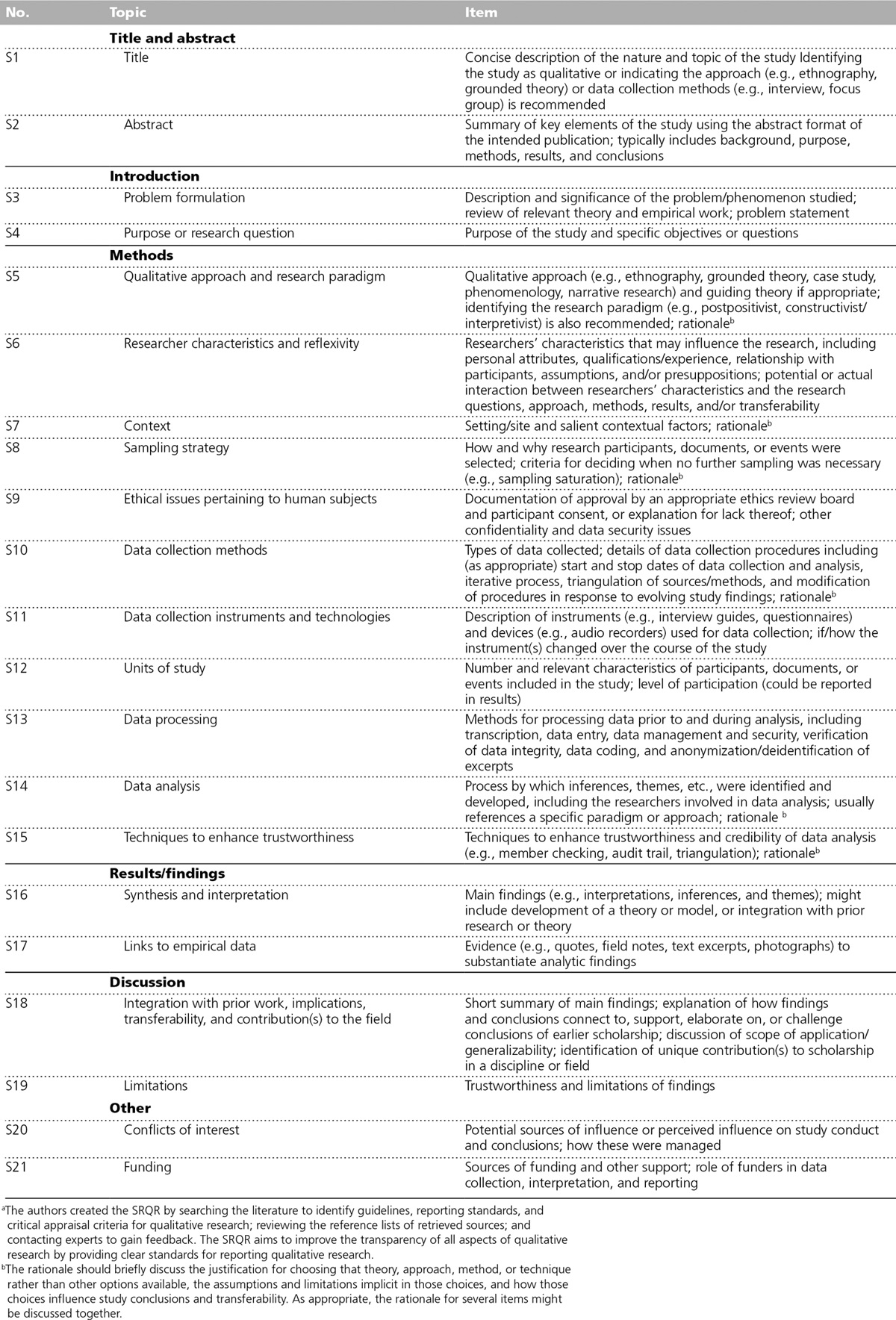
Page(s)**

1

1

2

2

2

2

2-3

2

2

2-3

2

3

2-3

2-3

2-3

3-5

3-5

5-6

6

8

7

**Reference**

1. O'Brien BC, Harris IB, Beckman TJ, Reed DA, Cook DA. Standards for reporting qualitative research: a synthesis of recommendations. *Academic medicine : journal of the Association of American Medical Colleges.* 2014;89(9):1245-1251.
